# VIPAS39 related arthrogryposis-renal dysfunction-cholestasis syndrome—case report and systematic review

**DOI:** 10.1186/s13023-024-03486-2

**Published:** 2024-12-30

**Authors:** Jan Kafol, Barbara Gnidovec Strazisar, Ana Drole Torkar, Matjaz Homan, Sara Bertok, Matej Mlinaric, Jaka Sikonja, Jernej Kovač, Mirjana Perkovic Benedik, Tanja Kersnik Levart, Mojca Zerjav Tansek, Marina Praprotnik, Tadej Battelino, Maruša Debeljak, Urh Groselj

**Affiliations:** 1 Faculty of Medicine, University of Ljubljana, Ljubljana, Slovenia; 2 Faculty of Medicine, University of Maribor, Maribor, Slovenia; 3 Department of Pediatrics, General Hospital Celje, Celje, Slovenia; 4 Department of Endocrinology, Diabetes, and Metabolic Diseases, University Children’s Hospital, University Medical Centre Ljubljana, Bohoriceva 20, 1000 Ljubljana, Slovenia; 5 Department of Gastroenterology, Hepatology and Nutrition, University Children’s Hospital, University Medical Center Ljubljana, Ljubljana, Slovenia; 6 Clinical Institute for Special Laboratory Diagnostics, University Children’s Hospital, University Medical Centre Ljubljana, Ljubljana, Slovenia; 7 Department of Child, Adolescent and Developmental Neurology, University Children’s Hospital, University Medical Centre Ljubljana, Ljubljana, Slovenia; 8 Department of Nephrology, University Children’s Hospital, University Medical Centre Ljubljana, Ljubljana, Slovenia; 9 Department for Pulmonary Diseases, University Children’s Hospital Ljubljana, University Medical Centre Ljubljana, Ljubljana, Slovenia

**Keywords:** Arthrogryposis–renal dysfunction–cholestasis syndrome, ARC syndrome, ARCS2, VIPAS39, VIPAR

## Abstract

**Background:**

Arthrogryposis–renal dysfunction–cholestasis (ARC) syndrome, a rare autosomal recessive disorder, exhibits genetic heterogeneity with the *VIPAS39* gene pathological variants being a distinct contributor.

**Results:**

We present two related patients from Kosovo, describing the clinical, genetic, and therapeutic aspects of the syndrome. The identified novel *VIPAS39* pathological variants (c.762G > A; c.1064_1082delinsAGTG) emphasize the complex phenotypic expression of ARC syndrome. A systematic literature review identified 8 *VIPAS39*-related ARC cases with notable variability in clinical features. Prognostically, patients fell into severe and milder groups, with some reaching adolescence. Our report aligns with others noting milder ARC courses and emphasizes the value of genetic testing, especially in atypical presentations. Challenges included incomplete literature data, early mortality affecting diagnostic workup, and limited *VIPAS39*-related ARC cases. Comparisons with the more prevalent *VPS33B* pathological variants revealed no distinct clinical differences.

**Conclusion:**

Our study expands understanding of ARC syndrome, highlighting its genetic diversity and clinical variability. Milder presentations underscore diagnostic challenges and the potential prevalence of undiagnosed cases. Increased awareness and comprehensive genetic testing are crucial for early and accurate diagnosis.

## Background

Arthrogryposis–renal dysfunction–cholestasis (ARC) syndrome is a rare autosomal recessive disorder, which was initially observed in consanguineous families. It is characterised by a classic triad of symptoms: arthrogryposis, renal dysfunction, and cholestasis [1].

Approximately 75% of ARC syndrome cases are caused by pathological variants in the vacuolar sorting–associated protein 33B (*VPS33B)—*ARC syndrome type 1 (ARCS1; Online Mendelian Inheritance in Man (OMIM): # 208,085 [2]). *VPS33B* plays a crucial role in intracellular protein sorting and vesicular trafficking, thus pathological variants in VPS33B result in multifaceted systemic dysfunction [3, 4]. Moreover, in 25% of cases of ARC syndrome, *VPS33B*-interacting protein *(VIPAS39;* also known as *VIPAR*) was identified as a causative gene—ARC syndrome type 2 (ARCS2; OMIM: # 613,404 [2]). There are only a few published reports on ARCS2, and clinical experiences are limited. Our knowledge and understanding of ARCS2 are mostly based on ARCS1 [5].

In addition to the classic array of symptoms, the clinical presentation of ARC syndrome often includes faltering growth and recurrent infections [1]. Common skin manifestations encompass ichthyosis and hyperkeratosis, along with dysmorphic features (large hands, proximally placed thumbs, low-set ears, high-arched palate, sloping forehead), and hirsutism. Additional features may include congenital heart defects, hypothyroidism, Anaemia, bleeding disorder due to abnormal platelet function, hearing loss, and corpus callosum agenesis [6, 7].

There are no accurate estimates of the ARC syndrome prevalence. In Western countries it is estimated to be less than 1 in a million, however, it is more prevalent in Saudi Arabia and Pakistan [8, 9].

In the absence of causal treatment, the prognosis for ARC syndrome is generally poor, with most patients not surviving beyond the first year of life [7, 8].

Due to the limited understanding of ARCS2, we aimed to present comprehensive clinical data on two ARCS2 patients with novel *VIPAS39* pathogenic variants from our centre. Additionally, we performed the first systematic review of all currently described cases with *VIPAS39-related* ARC syndrome or ARCS2 to summarize the clinical characteristics of the syndrome.

## Methods

### Case description

We collected the information from the electronic medical records of two patients, who were closely monitored and treated at the University Children’s Hospital, University Medical Centre Ljubljana. To ensure ethical considerations, written informed consent, utilizing local consent forms, was obtained from the parents of both patients. This consent was obtained for the potential publication of any identifiable images or data included in this article. CARE reporting guidelines were followed [10].

Diagnosis of ARC syndrome was established through a comparison of the patient’s clinical characteristics to similar cases in the literature and confirmed with genetic testing.

The UK-WHO charts were employed for computing percentiles/Z scores for anthropometric measurements. Standard methods were utilized to analyse laboratory measurements derived from blood and urine samples. Electrocardiogram, abdomen and heart ultrasonography, and esophagogastroduodenoscopy were conducted and interpreted by specialists in the respective field. We measured bone density with dual-energy X-ray absorptiometry (DXA).

To help us characterise the swallowing problems we used a barium swallow study.

A magnetic resonance imaging (MRI) of the brain was conducted to clarify the patient’s neurological symptoms. An electroencephalogram (EEG) was conducted to assess the electrical activity in the brain. Electrophysiological investigations included brainstem auditory evoked potentials for hearing screening and needle electromyography (EMG) to assess the function of muscles and peripheral nerves.

Thrombocyte aggregation was evaluated with Lumiaggregometry, additionally, the structure of thrombocytes was examined in peripheral blood smear.

Genetic analysis was performed in June 2023 on our two patients and their parents by next-generation sequencing (NGS). Regions of interest were enriched using the TruSight One library enrichment kit. The whole genome sequencing (WGS) for both patients was performed using NEBNext® Ultra™ II DNA Library Prep Kit for Illumina® (NEB, United States) according to the manufacturer's instructions and sequenced on the NovaSeq 6000 sequencer (Illumina, San Diego, CA, United States). A panel of genes associated with global developmental delay (HP: 0001263), including the *VIPAS39* gene, was used and filtered as a duo. In analysed genes, we achieved 45 times sequencing coverage of analysed regions in patient 1 and 53 times in patient 2. Family segregation for both parents was performed using Sanger sequencing.

### Systematic review of literature

**Fig. 1:**
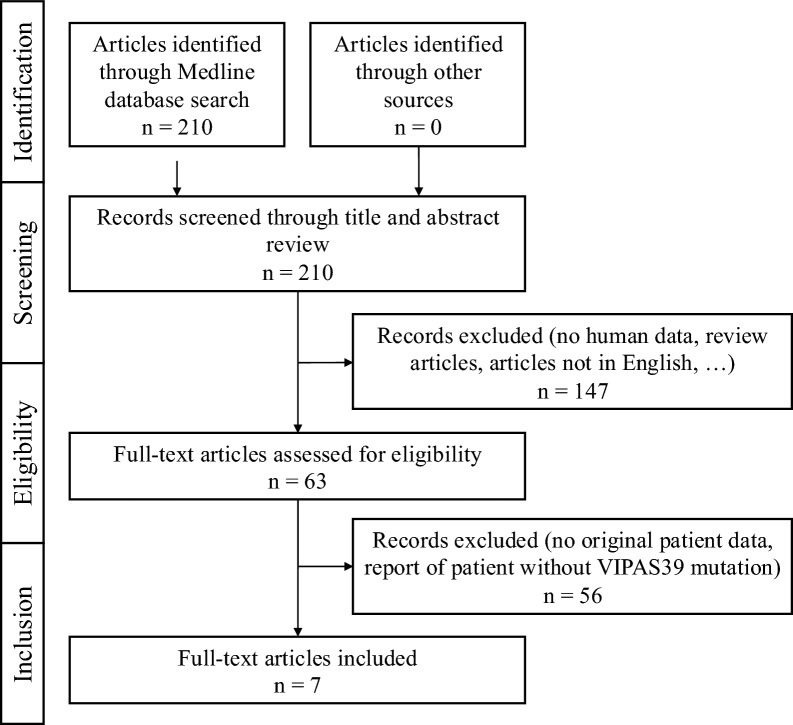
PRISMA flow diagram for systematic literature review

A systematic literature review was performed following PRISMA reporting guidelines (Fig. 1) on December 14th, 2023, to combine the data of all known patients with *VIPAS39* pathological variants and ARC syndrome [11]. We searched the Medline database for available case report articles on patients with pathogenic *VIPAS39* variants using the search terms “*VIPAS39*”, “*VIPAR*”, “*SPE-39*”, “*C14ORF133*”, “*VPS33B*”, “ARC syndrome”, “arthrogryposis renal dysfunction cholestasis” and identified 210 different research articles. In addition, we performed the inspection of references of the identified articles, however, this did not identify any additional relevant articles. During the screening process of article titles and abstracts, we excluded all that did not report human data, articles that did not match the desired topic, review articles, and articles not available in English. The remaining 63 articles were assessed thoroughly for eligibility. We decided to focus only on *VIPAS39* pathological variants since there are already published reviews of *VPS33B* cases [7, 8, 12]. We included 7 articles that reported original patient data (Fig. 1).

## Results

### Case presentation 1

Patient 1, a female, was born vaginally 10 days after term following an uneventful pregnancy in Kosovo. Her Apgar's score in the 1st minute was 7, and she required short ventilation support using a breathing mask. Since birth, she experienced itchy skin and cough.

**Fig. 2:**
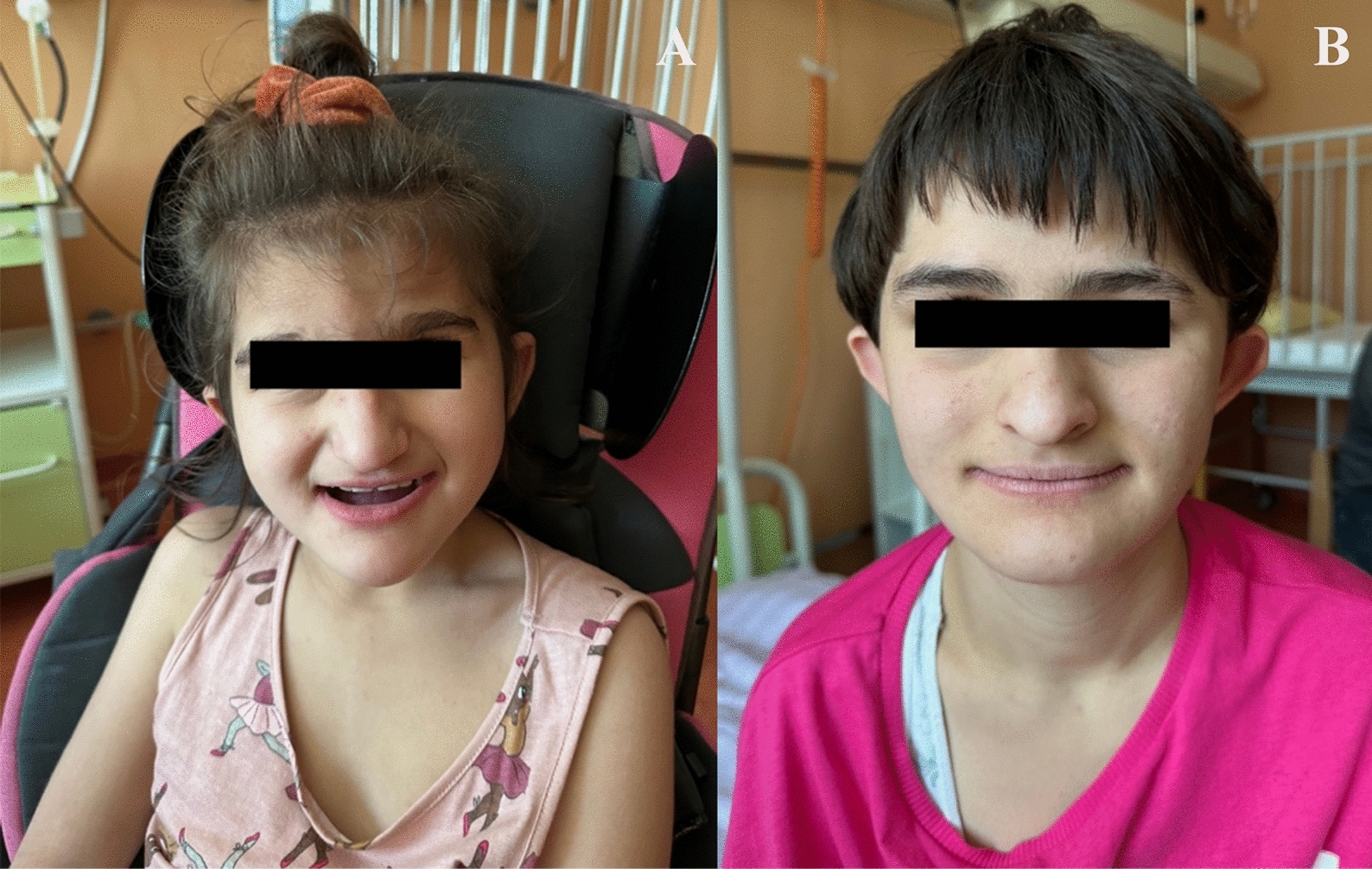
Patient 1 **A** and Patient 2 **B**, both displayed dysmorphic features (flat occiput, triangular-shaped face, larger protruding ears, a lower hairline, mild hypertelorism)

The patient was hospitalized for acute bronchiolitis and pneumonia at the age of 2.5 years at the University Children’s Hospital Ljubljana, where examination revealed agitation, ichthyosis, dry and cracked skin due to constant scratching, dysmorphic features (Fig. 2A), and moderate virilization of the genitalia, characterized by a prominent clitoris. There were no signs of arthrogryposis. Pronounced developmental intellectual disability with microcephaly was evident. She was hypotonic with reduced tendon reflexes and distal muscle strength, however, needle EMG with nerve conduction study revealed normal results. EEG showed slow and irregular background activity without any paroxysmal discharges. On brain MRI profound corpus callosum hypoplasia with cerebellar tonsillar ectopia was seen.

There was growth delay in infancy and early childhood, which showed improvement following nutritional intervention upon the diagnosis of moderate malnutrition at the age of 3 years. However, despite nutritional support, the patient did not achieve the anticipated body growth—at the age of 10 years, she was 117 cm (< 1st percentile) tall and weighed 23.8 kg (1st percentile).

The patient had elevated liver enzymes throughout the follow-up period. At the age of 5, her laboratory profile showed elevated aspartate aminotransferase (AST) 1.22 µkat/L (reference range (RR): < 0.78) and alanine aminotransferase (ALT) 1.72 µkat/L (RR: < 0.43), in addition to normal gamma-glutamyl transferase (GGT) 0.24 µkat/L (RR: 0.06—0.37). Furthermore, alkaline phosphatase (ALP) was significantly increased at 9.90 µkat/L (RR: 1.60—4.96). However, total bilirubin levels remained within the normal range—4 µmol/L (RR: < 17), as well as serum albumin—37 g/L (RR: 32—55). Coagulation tests were normal: prothrombin time (PT) 9.6 s (RR: 12.1—14.5), international normalized ratio (INR) 0.78 (RR: 0.92—1.14), activated partial thromboplastin time (aPTT) 28 s (RR: 33.6—43.8). Ultrasound of the abdomen was unremarkable.

At the age of 2 years and 7 months, tubulopathy characterized by proteinuria (0.43 g (42 mg/kg/m2) in 24 h urine collection) was noted, and proteinuria persisted throughout the follow-up period. Kidney ultrasound revealed reduced corticomedullar differentiation and uneven heterogenous reflections in pyramids.

The genetic analysis revealed that patient 1 and her sister (patient 2) were compound heterozygous for two pathological variants in the *VIPAS39* gene. The first variant NM_001193314:c.762G > A resulted in the alteration of the last nucleotide in exon 11 and affected intron excision (p.Ala254 =). The second variant NM_001193314:c.1064_1082delinsAGTG resulted in the deletion of 6 amino acids, followed by the insertion of glutamine and an early termination codon (p.Pro355_Thr361delinsGlnTer). Both variants were not previously reported in the Human Gene Mutation Database (HGMD®) [13], ClinVar [14], and healthy populations, and they were considered likely pathologic (PVS1, PM2) according to the American College of Medical Genetics and Genomics (ACMG) criteria [15]. Described variants caused ARCS2, which is inherited autosomal recessively. The first variant was inherited from patients’ mother and the second from their father.

Following the genetic confirmation of ARC syndrome, a thrombocyte morphological analysis revealed the absence of alpha granules and functional tests showed aggregation dysfunction.

In the absence of specific treatment, the patient was managed symptomatically. At the age of 10 years and 10 months, she was hospitalized due to viral pneumonia caused by influenza A virus. Treatment with oseltamivir was initiated, and as bacterial coinfection was suspected, antibiotic treatment was also started. Despite the dual treatment mentioned above, she passed away.

### Case presentation 2

Patient 2, an older sister of patient 1, was born after an uneventful pregnancy via Caesarean section. She underwent an initial examination in our medical centre by a paediatric neurologist at the age of 8, when she presented as pale, with stunted growth, arthrogryposis, ichthyosis, and dysmorphic features (Fig. 2B).

She experienced global developmental delay and attended a special needs school. Adequate muscular tonus and strength were observed with reduced tendon reflexes, however, EMG returned normal results. EEG showed potential abnormalities due to irregular background activity with some focal slowing. No seizures were observed. Brain MRI showed corpus callosum hypoplasia with cerebellar tonsillar ectopia.

Orthopaedic issues included bilateral hip displacement and congenital equinovarus deformity requiring early surgical intervention with subsequent slight leg length discrepancy with shorter left leg and foot.

Liver tests at the age of 16 years were within normal range: AST 0.60 µkat/L (RR: < 0.61); ALT 0.48 µkat/L (RR: < 0.52); GGT 0.21 µkat/L (RR: 0.06—0.70); total bilirubin 8 µmol/L (RR: < 17); Coagulation tests were normal: PT 10.3 s (RR: 12.7—16.1), INR 0.88 (RR: 0.97—1.30), aPTT 26 s (RR: 33.9—46.1). Ultrasound of the abdomen was unremarkable.

At the age of 8.5 years, a laboratory test detected proteinuria, which was not detectable in follow-up tests. Therefore, tubulopathy was ruled out. Additionally, an ultrasound of the urinary system showed no anomalies.

The patient did not reach anticipated growth—measuring at 143 cm (< 1st percentile) and weighing 46.6 kg (9th percentile) at the age of 16 years.

Genetic analysis revealed the same alterations of the *VIPAS39* gene as described above for her younger sister (patient 1), confirming the diagnosis of ARCS2.

Following the confirmed diagnosis of ARC syndrome, Lumiaggregometry revealed aggregation dysfunction due to abnormalities in blood platelet function.

The patient received only symptomatic management and was still alive at the time this report was written. Additionally, the patient had a younger healthy brother who was three years younger.

Clinical characteristics of both presented patients are collected in Table 1.

**Table 1:** Clinical characteristics of patients 1 and 2

Parameter	Patient 1	Patient 2
Birth Details	Vaginal birth, uneventful pregnancy	Caesarean section, uneventful pregnancy
	Birth measurements: 3940 g, 51 cm, 36 cm	Birth measurements: 3550 g, 51 cm, 34 cm
	Ventilation required at birth	
Dermatological issues	Ichthyosis, plantar hyperkeratosis	Ichthyosis, hair-pulling, and plantar hyperkeratosis
Dysmorphic features	Flat occiput, triangular-shaped face, larger protruding ears with a simplified helix, a lower hairline, mild hypertelorism	Triangular-shaped face, lower hairline, protruding ears, simplified helix, mild hypertelorism
Orthopaedic Issues	Short and thick fingers, and pectus excavatum	Pes equinovarus, bilateral hip dislocation in infancy; short and thick fingers, shorter left leg
Neurological Findings	Global developmental delay with profound intellectual disability, acquired microcephaly, reduced muscular tone, unable to sit up to the age of 6, after that wheelchair-bound (progressed to crawling), nonverbal, could not control urine and faeces. Reduced tendon reflexes with reduced strength in distal lower extremities (EMG normal)	Global developmental delay with severe intellectual disability—first words at 3,5 years, started walking at 5; communication involved syllables, few words, she followed given instructions; Generalized hypotonia with reduced tendon reflexes and proximal weakness in lower extremities (EMG normal)
	Brain MRI: hypoplasia of the corpus callosum and anterior commissure, cerebellar tonsillar ectopia; EEG: abnormal-irregular and slower background activity	Brain MRI: corpus callosum hypoplasia, cerebellar tonsillar ectopia; EEG: abnormal-irregular background activity with focal slowing
Eye and Ear Conditions	Eye surgery at age 8 for cataracts	Normal eye examination
	Bilateral deafness was confirmed at 3 years, and 6 months	
Gastrointestinal Issues	Feeding via gastrostomy tube, inserted at 2 years and 11 months, due to uncoordinated swallowing (confirmed with the barium swallow study)	Looser stools, lactose intolerance
	Reflux esophagitis (confirmed with esophagogastroduodenoscopy)	
Liver Function	Elevated liver enzymes	Elevated liver enzymes
Heart function	No abnormalities on electrocardiogram and ultrasound	No detected abnormalities
Kidney function	Tubulopathy	Normal function
Growth and Nutrition	Growth delay, and malnutrition diagnosed in early childhood	Growth delay, decreased appetite until age 5
	DXA revealed reduced bone density (Z: -1.1), but there were no fractures	Regular periods, puberty status reached
Platelet Function	Absence of alpha granules, aggregation dysfunction	Aggregation dysfunction
Other Issues	Microcytic anaemia	Microcytic anaemia
	At 2,5 years hospitalized for bronchiolitis and pneumonia At 3 years and 9 months acute respiratory failure due to the aspiration of blood, which was triggered by the inability to control bleeding after the extraction of 20 deciduous teeth At almost 11 years acute respiratory failure and death due to severe pneumonia	

### Systematic review of literature

**Fig. 3:**
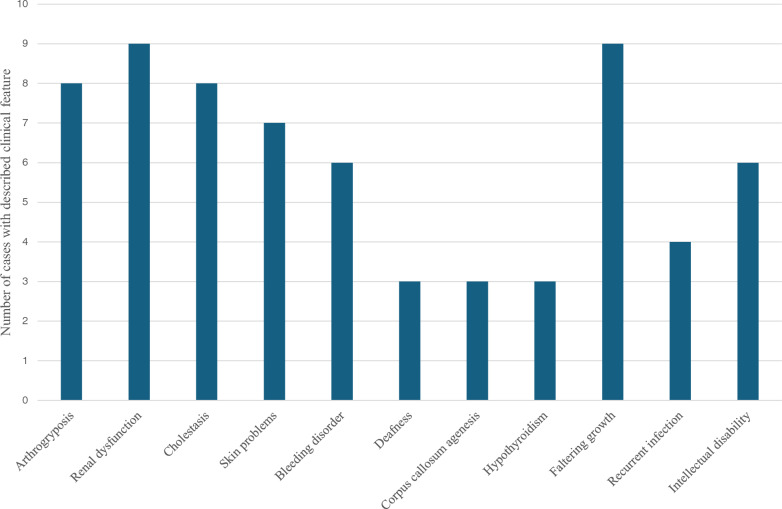
Common clinical features of reported ARCS2 cases

We have identified 8 reported cases of ARCS2 in the literature. The genetic and clinical characteristics of 10 patients with *VIPAS39* pathological variants are summarised in Table 2 and displayed on the graph in Fig. 3. Homozygous patients appeared, with one exception, due to consanguinity. In contrast, parents of heterozygous patients were not closely related. In nearly every patient, the disease manifested with the classic triad of symptoms: arthrogryposis, renal dysfunction, and cholestasis with normal levels of GGT. Notably, faltering growth emerged as the most common non-classic clinical feature (9/10). Platelet dysfunction or bleeding disorders were often described (7/10). Skin manifestations, such as ichthyosis or hyperkeratosis were also common (7/10). Neurological abnormalities were observed, including intellectual disability (6/10), generalized hypotonia, bilateral deafness, and corpus callosum agenesis (3/10). Laboratory tests revealed hypothyroidism in 3 patients, with an additional report of anaemia in one instance. Prognostically, patients could be differentiated into two groups: one with a very poor prognosis, with a lifespan under 1 year, and a second group with a milder disease presentation and noticeably better survival, with individuals reaching adolescence. The longest-living patient was still alive at the age of 24 years [9].

**Table 2:** A list of reported cases of ARC syndrome with VIPAS39 pathological variants so far

Patient	Reference	Mutation sites	Homozygotes/CH	Amino acid change	Country of origin	Arthrogryposis	Renal dysfunction	Cholestasis	Ichthyosis/hyperkeratosis	Platelet dysfunction/bleeding disorder	Sensorineural deafness
I	[9]	339del; 1035C>G	CH	Phe113Leufs*60; Tyr345*	Japan	−	+	+	+	−	+
II	[22]	NA	Hom	NA	India	+	+	+	+	−	NA
III	[23]	618_626dup	Hom	Arg206_Leu208dup	India	+	+	+	NA	NA	NA
IV	[24]	63_464delTG; 484C>T	CH	Trp155GlufsX4; Arg162X	United Kingdom	+	+	+	+	+	NA*
V	[24]	808C>T	Hom	Arg270X	Pakistan	+	+	+	+	+	NA*
VI	[25]	484C>T	Hom	Arg162Stop	NA	+	+	+	NA	+	NA
VII	[26]	1130G>C	Hom	Arg377Pro	Iran	+	+	+	NA	NA	+
VIII	[27]	1021T>C	Hom	Cys341Arg	Pakistan	+	+	+	+	+	
IX	Our patient 1	762G>A; 1064_1082delinsAGTG	CH	Ala254=; Pro355_Thr361delinsGlnTer	Kosovo	−	+	−	+	+	+
X	Our patient 2	762G>A; 1064_1082delinsAGTG	CH	Ala254=; Pro355_Thr361delinsGlnTer	Kosovo	+	−	−	+	+	−

Patient	Reference	Mutation sites	Homozygotes/CH	Amino acid change	Country of origin	Agenesis of the corpus callosum	Hypothyroidism	Faltering growth	Recurrent infection	Intellectual disability	Survival
I	[9]	339del; 1035C>G	CH	Phe113Leufs*60; Tyr345*	Japan	NA	NA	+	NA	+	Alive at 24 years
II	[22]	NA	Hom	NA	India	NA	+	+	+	+	NA
III	[23]	618_626dup	Hom	Arg206_Leu208dup	India	NA	NA	+	NA	NA	NA**
IV	[24]	63_464delTG; 484C>T	CH	Trp155GlufsX4; Arg162X	United Kingdom	NA	+	+	+	+	9 months
V	[24]	808C>T	Hom	Arg270X	Pakistan	NA	−	+	+	+	20 months
VI	[25]	484C>T	Hom	Arg162Stop	NA	NA	NA	+	−**	NA	Alive at 3 months
VII	[26]	1130G>C	Hom	Arg377Pro	Iran	NA	+	NA	NA	NA	Alive at 2,5 months
VIII	[27]	1021T>C	Hom	Cys341Arg	Pakistan	+	NA	+	NA	NA	7 months
IX	Our patient 1	762G>A; 1064_1082delinsAGTG	CH	Ala254=; Pro355_Thr361delinsGlnTer	Kosovo	+	NA	+	+	+	10 years and 10 months
X	Our patient 2	762G>A; 1064_1082delinsAGTG	CH	Ala254=; Pro355_Thr361delinsGlnTer	Kosovo	+	−	+	−	+	Alive at 16 years

*NA* Not available

*CH* Compound heterozygotes

*The article states that two out of three described patients had sensorineural deafness but does not specify which two patients

**Patient’s age at the time of report was 27 days

## Discussion

We described two new patients from Kosovo with ARC syndrome and identified novel heterozygous pathological variants of *VIPAS39* and conducted a systematic review of reported ARCS2 cases.

According to the ClinVar: public archive of relationships among sequence variation and human phenotype (accessed on February 19th, 2024), there have been 385 reported public variants of the VPS33 gene and 230 *VIPAS39* variants. Among the *VIPAS39* variants, 40 were classified as pathogenic or likely pathogenic, and out of those, 14 variants were linked to ARC syndrome. This further emphasizes the exceptional rarity of ARC syndrome resulting from the *VIPAS39* pathological variants [14].

The *VPS33B*, located in the 15q26.1 region, encodes the *VPS33B*, a homolog of the yeast class C vacuolar sorting protein Vps33p. Vps33p is a member of the Sec-1/Munc18 family. It interacts with soluble N-ethylmaleimide-sensitive factor attachment protein receptors (SNAREs), which are involved in synaptic transmission and vesicular secretion through mediating membrane targeting and fusion. Class C vps proteins compose homotypic fusion and vacuole protein sorting (HOPS) and class C core vacuole/endosome tethering (CORVET) complexes, that have a key role in the vesicular trafficking pathway [3, 16]. Pathological variants in *VPS33B* may cause disturbance in localisation and accumulation of plasma proteins in polarised kidney and liver cells, leading to a complex systemic impairment [8]. *VIPAS39* (*VIPAR*) is located in the 14q24.3 region. *VIPAS39* is composed of a golgin A5 domain and exhibits substantial homology with the C-terminal region of Vps16. Together with *VPS33B*, it forms a complex (*VPS33B-VIPAR*) that interacts with the RAB11A-recycling pathway that is involved in apical membrane protein sorting. *VPS33B-VIPAR* also regulates the expression of E-cadherin. *VPS33B-VIPAR* is involved in regulating apical-basolateral polarity in the liver and kidney [5, 17]. *VPS33-VIPAR* complex also participates in platelet alpha granule formation, therefore the absence of alpha granules in platelets can be observed in ARC syndrome [18].

Arthrogryposis in ARC syndrome may manifest as muscle atrophy, radial deviation of the wrist, bilateral dislocation of hips, flexion contracture of the knees, and calcaneovalgus. Cholestasis presents as jaundice without biliary obstruction, with variable bilirubin levels, and normal GGT, while AST and ALT may be normal or slightly elevated. Renal tubular dysfunction commonly appears in the form of Fanconi syndrome [7, 8].

Patients 1 and 2 exhibited a milder clinical presentation of ARC syndrome because they didn’t develop a full clinical picture of ARC syndrome, yet notable differences existed between both patients in the number and severity of symptoms, as well as in achieving developmental milestones.

Patient 1 primarily presented with global developmental delay with microcephaly, ichthyosis, and renal dysfunction manifesting as tubulopathy. The absence of cholestasis and arthrogryposis delayed the diagnosis. Interestingly the patient also developed bilateral cataract, which is an additional symptom, that so far has not been associated with ARC syndrome.

In patient 2, reduced tendon reflexes and the proximal weakness in lower extremities initially guided us toward the exclusion of neuromuscular disorders. Patient 2 achieved further development in comparison with patient 1. Kidney and liver function were normal, and the most prominent signs associated with ARC syndrome in patient 2 were arthrogryposis, ichthyosis, and platelet dysfunction. The difference in clinical presentation in each patient highlights the variability in the expressivity of the same *VIPAS39* pathological variants.

Diagnosis of ARC syndrome relies on the clinical presentation of the classic triad of symptoms. Pathological confirmation through biopsy is discouraged due to the serious risk of fatal bleeding complications, instead, genetic sequencing of *VPS33B* and *VIPAS39* is a much safer alternative [4, 8].

The diagnosis of ARC syndrome in our two cases was delayed due to their atypical presentation. Neither patient exhibited the full classic triad of symptoms, and the primary guideline in the diagnosis was global developmental delay. This stands in contrast to other published ARCS2 cases, where the triad was present in 7 out of 8 instances. Furthermore, while prolonged survival was rare in those cases, both of our patients exceeded the life expectancy associated with ARC syndrome. Notably, only one published report on ARCS2 mentioned corpus callosum agenesis, a feature present in both of our patients. Regarding similarities with previously published cases, the predominant issues reported, which we also observed in two of our patients, encompass skin problems, faltering growth, and intellectual disability.

Our two cases illustrate that ARC syndrome could potentially be more prevalent due to a higher number of undiagnosed atypical cases. We also demonstrated the importance of genetic testing to find the correct diagnosis in instances of rare diseases in the paediatric population. It is essential that a detailed clinical picture informs and guides the genetic diagnosis.

Our report on a milder course of ARC syndrome is not an isolated instance. Zhu et al. have compiled reports on 19 patients with milder ARC syndrome, leading to longer survival. Except for one subject with a *VIPAS39* pathological variant, all other patients had *VPS33B* pathological variants. They found that missense mutations were frequent in patients with ARC syndrome, who had longer survival. Milder presentation was associated with specific pathological variants of *VPS33B*, often resulting in only a partial loss of function [12]. In our case, the first pathological variant of *VIPAS39* affected only the intron excision, which may have resulted in residual VIPAS39 protein function, which would explain the milder presentation of ARCS2.

Yu et al. also reported a delayed diagnosis attributed to the absence or delayed onset of characteristic symptoms of ARC syndrome. In their study, a patient was diagnosed at the age of 13 years due to the absence of kidney dysfunction and the delayed onset of jaundice. Clinicians initially failed to recognize that ichthyosis and arthrogryposis were part of the ARC syndrome, a situation very similar to patient 2 in our case. In contrast, patient 1 did not present with arthrogryposis and cholestasis, only tubulopathy. These vast differences in clinical presentation further underscore the complexity of the disorder and the challenges in early diagnosis. In the same article, the authors also speculated that the prolonged lifespan may be attributed to mild symptoms, which can be managed symptomatically [19].

Both of our patients showed skin involvement, underscoring the importance of considering dermatological signs like ichthyosis or hyperkeratosis in VIPAS39-related ARC syndrome. In a review of the literature, skin manifestations were reported in 7 out of 10 cases, with the others lacking sufficient data—possibly due to limited focus on dermatology. The "ARC" acronym, emphasizing the primary symptoms of arthrogryposis, renal dysfunction, and cholestasis, can be misleading, leading clinicians to overlook other features like skin involvement. Thus, ARC syndrome should be viewed as a spectrum of presentations rather than a condition with fixed characteristics.

In the absence of causal treatment of ARC syndrome, symptomatic and supportive treatment strategies include fluid supplementation, infection management, enteral nutrition, ursodeoxycholic acid, fat-soluble vitamins, calcium glubionate, L-thyroxine, and phosphate [8, 12]. Surgery is often necessary to alleviate orthopaedic manifestations but should be avoided if long-term survival is not anticipated [20]. Liver transplant may be considered in cases of severe cholestasis and pruritus [21]. An essential aspect of the therapeutic approach is genetic counselling and prenatal genetic testing for families experiencing ARC syndrome [8].

Our literature review encountered limitations stemming from the completeness of published reports on ARC syndrome patients with *VIPAS39* pathological variants. Some reports lacked crucial data regarding the complete clinical picture of the patients. Moreover, these reports were composed early in the patients' lives, lacking information on outcomes, and making it challenging to assess the severity of the disease presentation. The premature mortality of some patients further hindered a comprehensive diagnostic workup; for example, developmental delay was not described in cases where patients didn’t survive past the first year.

We encountered challenges in gathering specific pathological variants information for each patient. The rarity of the *VIPAS39* pathological variants contributed to a small number of reported cases, in stark contrast to the more prevalent *VPS33B* pathological variants, where a sufficient number of cases offered better insight into the clinical impact [7]. Notably, Cullinane et al. found no observable difference in symptomatic presentation and disease course between subjects with *VIPAS39* and *VPS33B* pathological variants [5]. Our review similarly failed to identify any distinct clinical characteristics that would differentiate the mentioned pathological variants.

## Conclusion

Our study delves into the clinical presentation and genetic aspects associated with ARC syndrome. Through a systematic literature review and the presentation of two new cases, we expanded the knowledge of ARCS2. We more precisely characterized the clinical features of the syndrome, encompassing not only the classic triad of symptoms but also skin manifestations, neurological issues, developmental delay, faltering growth, bleeding disorders, hypothyroidism, and recurrent infections. While the severity of symptoms and survival may be influenced by the type of pathological variant and the residual function of the VIPAS39 protein, our findings demonstrate that these factors do not consistently predict phenotype, highlighting the lack of a clear genotype–phenotype correlation.

## Data Availability

The raw data supporting the findings of this study are available in the Mendeley Data repository: Kafol, Jan (2024), “VIPAS39 related arthrogryposis-renal dysfunction-cholestasis syndrome—case report and systematic review”, Mendeley Data, V2, DOI: 10.17632/J65t6xdmmv.2. Access to detailed data is restricted to protect individuals' privacy under the European General Data Protection Regulation (GDPR).

## Abbreviations

ALP: Alkaline Phosphatase
ALT: Alanine Aminotransferase
ARC syndrome: Arthrogryposis–renal dysfunction–cholestasis syndrome
ARCS1: ARC syndrome type 1
ARCS2: ARC syndrome type 2
ACMG: American College of Medical Genetics and Genomics
AST: Aspartate Aminotransferase
DXA: Dual-energy X-ray Absorptiometry
EEG: Electroencephalogram
EMG: Electromyography
GGT: Gamma-Glutamyl Transferase
HGMD: Human Gene Mutation Database
HOPS: Homotypic Fusion and Vacuole Protein Sorting
INR: International Normalized Ratio
MRI: Magnetic Resonance Imaging
NGS: Next-generation Sequencing
OMIM: Online Mendelian Inheritance in Man
PRISMA: Preferred Reporting Items for Systematic Reviews and Meta-Analyses
PT: Prothrombin Time
RR: Reference Range
VIPAS39: VPS33B-interacting protein
VPS33B: Vacuolar Sorting–Associated Protein 33B
WGS: Whole Genome Sequencing
